# Effects of Imaging Parameters on the Quality of Contrast-Enhanced MR Angiography of Cerebral Aneurysms Treated Using Stent-Assisted Coiling: A Phantom Study

**DOI:** 10.2463/mrms.mp.2016-0042

**Published:** 2016-09-06

**Authors:** Yoichiro Ikushima, Takashi Hashido, Yoshiyuki Watanabe, Tsukasa Doi

**Affiliations:** 1 Department of Medical Technology, Osaka University Hospital, 2-15 Yamadaoka, Suita, Osaka 565-0871, Japan; 2 Department of Radiology, Graduate School of Medicine, Osaka University, Suita, Japan

**Keywords:** contrast-enhanced MRA, imaging parameter, stent-assisted coiling

## Abstract

**Purpose:**

To quantitatively investigate *in vitro* the effects of flip angle (FA), receiver bandwidth (BW), echo time (TE), and magnetic field strength (FS) on image noise and artifacts induced by stent-assisted coiling on contrast-enhanced MR angiography (CE-MRA) images, as a first step towards optimization of imaging parameters.

**Methods:**

A phantom simulating a cerebral aneurysm treated using stent-assisted coiling was filled with diluted gadolinium contrast medium, and MR angiography were obtained using varied parameters: FA (10°–60°), BW (164–780 Hz/pixel), and FS (1.5 and 3.0T). The TE varied automatically with BW because the TE was set to the smallest value. Three kinds of indices were semi-automatically calculated to quantify the severity of stent- and coil-induced artifacts: artificial lumen narrowing (ALN) representing a decrease in the in-stent luminal area, and relative in-stent signal (RIS_S_) and relative in-coil signal (RIS_C_) representing an increase in the in-stent and in-coil signal intensities, respectively. We also measured the ratio of in-stent signal to noise (IS/N) for each parameter. The variation in these indices with variations in FA, BW (TE), and FS was analyzed.

**Results:**

An increase in FA led to an increase of up to 65% in the RIS_S_, while the IS/N increased by up to three times. The 1.5T scanner indicated fewer artifacts (71% lower ALN, two times higher RIS_S_, and 40% higher RIS_C_) than the 3.0T scanner. On the other hand, the 1.5T scanner worsened the IS/N compared with the 3.0T scanner, although the difference was relatively small. Variation in BW (and hence, TE) led to a trade-off between artifact severity and IS/N.

**Conclusion:**

A high FA and low FS should be used for improved artifact severity and IS/N on CE-MRA images of a stent-assisted coil. A wide BW (short TE) could improve artifact severity at the expense of the image noise.

## Introduction

Stent-assisted coiling is one of the treatments for a cerebral aneurysm with a wide neck.^1^ Because of the possibility of restenosis of the stent lumen and a remnant aneurysm flow associated with this treatment, follow-up examination is required.^2,3^ Digital subtraction angiography and magnetic resonance angiography (MRA) are frequently used for follow-up examination. MRA has the advantage of minimal invasiveness and is free from ionizing radiation. Contrast-enhanced MRA (CE-MRA) is reported to be a better technique for follow-up compared with time-of-flight MRA in terms of depiction of the parent artery lumen and aneurysm neck.^4^ However, the delineation of the vessel lumen on CE-MRA images can be degraded by artifacts induced by the metallic stent and coil. We need to optimize the imaging parameters of CE-MRA in order to minimize artifacts. Several *in vitro* studies on the optimal parameters for CE-MRA for either a stent or coil have been documented.^5–7^ However, to our knowledge, there has been no phantom study on optimized parameters of CE-MRA for both a stent and coil. The artifacts induced by the stent and coil comprise mainly the susceptibility artifact and radiofrequency (RF)-shielding artifact.^7,8^ The severity of the susceptibility artifact is known to be decreased by several factors including short echo time (TE), wide receiver bandwidth (BW), and low magnetic field strength (FS).^9^ The severity of the RF-shielding artifact is decreased by a high flip angle (FA) and low FS.^10,11^ However, the wide BW and low FS that decrease artifacts can lead to relatively noisy images. Therefore, it is important to quantify the effects of these parameters on the image noise and the stent- and coil-induced artifacts.

The purpose of this study was to investigate *in vitro* the effects of FA, FS, BW, and TE on image noise and artifacts induced by a stent-assisted coil in CE-MRA, as a first step towards determining the optimal imaging parameters for minimizing artifacts without compromise of the image noise.

## Materials and Methods

Our study did not require institutional review board approval because no patients or animals were scanned and no clinical images were used.

### Acquisition of MRA images of a tubular phantom

**Fig 1.**
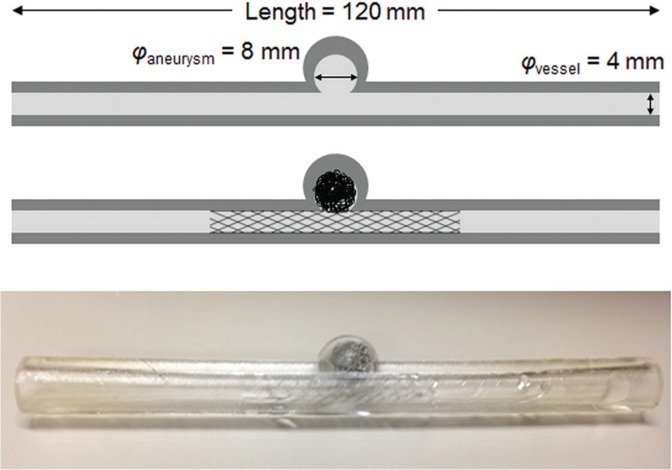
The schemes of the original phantoms simulating a vessel with a cerebral aneurysm: the reference phantom (without any device) in this study (Top) and the experimental phantom (with a stent and coil) (Middle), and a picture of the experimental phantom (Bottom). These phantoms were made of silicon.

We prepared two original tubular phantoms simulating a vessel with a cerebral aneurysm (Fig. 1). One of the phantoms was with a coil (Orbis, 0.9 mm × 25 cm, made of platinum, Codman) and a stent (Enterprise, 28 mm × 4.5 mm, made of nitinol, Cordis), and the other was without these. The phantom without these devices was regarded as reference in this study. These phantoms were filled with 5 mm gadolinium contrast medium (Magnevist, Bayer Healthcare, Osaka, Japan), set parallel to each other in a plastic box, and fixed with agarose gel (T_1_ = 828 ms at 3.0 T). The concentration of 5 mM corresponded to that of the aorta when administering 3 ml/s,^12^ and T_1_ of 828 ms corresponded to that of the parenchyma of the brain.^13^

A 1.5T MR scanner (Ingenia 1.5T, Philips Medical Systems, Best, The Netherlands) with an 8-channel base coil and a 7-channel head coil, and a 3.0T scanner (Achieva 3.0T, Philips Medical Systems, Best, The Netherlands) with a 32-channel head coil were used in this study. The plastic box containing the two phantoms was placed at the center of the receiver coil. The long axis of the phantoms was parallel to the direction of the static MF. Transverse MRA images of the box were acquired using three-dimensional T_1_-weighted fast field echo sequence. FA and BW were varied at 6 levels (10°–60°) and 7 levels (164–760 Hz/pixel), respectively (Table 1), and each scan was repeated consecutively two times. The image series acquired using the 1^st^ and 2^nd^ scans were defined as the series A and B, respectively. A total of 52 image series ([6 FAs + 7 BWs] × 2 times × 2 scanners) were acquired. Note that the TE and repetition time (TR) automatically changed with the BW (depended on by TE and TR) and FA (depended on by only TR) because they were set at “Shortest”, in accordance with the settings of CE-MRA in clinical practice. FA was fixed at 60° when BW was varied and BW was fixed at 634 Hz/pixel when FA was varied, because these fixed FA and BW values were empirically assumed to provide the best image quality (regarding the artifact and image noise) in this study. The other parameters were fixed as follows: field of view, 220 mm × 198 mm; acquisition matrix, 256 × 230; reconstruction matrix, 512 × 460; slice thickness, 0.4 mm (after zero-fill interpolation); number of slices, 140; with half scan; and profile order, CENTRA.

### Quantification of artifacts and image noise

**Fig 2.**
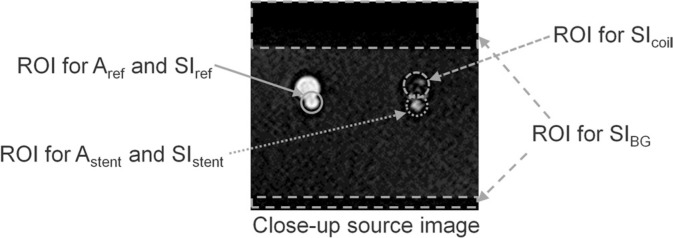
Locations of the regions of interest (ROIs) used for measurements of the luminal areas (A) and the signal intensities (SI).

Three kinds of indices were used to quantify the severity of artifacts: artificial lumen narrowing (ALN),^5,14,15^ relative in-stent signal (RIS_S_),^6,15^ and relative in-coil signal (RIS_C_). The ALN indicated the severity of the artifact that decreased the apparent luminal area inside a stent, using the following equation:

(1) $$\text{ALN}=\frac{{\text{A}}_{\text{ref}}-{\text{A}}_{\text{stent}}}{{\text{A}}_{\text{ref}}}$$

where A_stent_ and A_ref_ indicate the apparent luminal areas inside the stent adjacent to the coil and inside the phantom without devices (reference phantom), respectively (Fig. 2). These “apparent luminal areas” were defined as the number of pixels having signal intensity (SI) higher than half maximum SI of the lumen (a region of interest, Fig. 2) in this study, and were semi-automatically measured by means of our originally developed software that was written in the C language. An ALN value closer to 0.0 meant fewer artifacts (larger area), and that is closer to 1.0 meant more artifacts. We also estimated the luminal diameter inside the stent ( $=2\sqrt{{\text{A}}_{\text{stent}}/\pi }$ ) for intuitive understanding of the artifact severity.

The RIS_S_ and RIS_C_ indicated the severity of the artifact that decreased the SI inside a stent and inside a coil, respectively, and were calculated using Eqs. (2) and (3):

(2) $${\text{RIS}}_{\text{S}}=\frac{{\text{SI}}_{\text{stent}}-{\text{SI}}_{\text{BG}}}{{\text{SI}}_{\text{ref}}-{\text{SI}}_{\text{BG}}}\times 100(\%)$$

(3) $${\text{RIS}}_{\text{C}}=\frac{{\text{SI}}_{\text{coil}}-{\text{SI}}_{\text{BG}}}{{\text{SI}}_{\text{ref}}-{\text{SI}}_{\text{BG}}}\times 100(\%)$$

where SI_stent_, SI_coil_, SI_ref_, and SI_BG_ indicate the SIs inside the stent adjacent to the coil, inside the coil, inside the reference phantom and that of the background area (consisting of air), respectively, on the CE-MRA image (Fig. 2). These SIs were semi-automatically measured by means of the original software. RIS_S_ and RIS_C_ values closer to 100% indicated fewer artifacts (higher SI inside the stent and coil), and those closer to 0% indicated more artifacts. RIS_C_ was our original index and a lowered RIS_C_ could worsen the detectability of the aneurysmal remnant.

The RIS_S_ was an index for evaluating the SI inside the stent without consideration of image noise. The image noise could vary with several factors including BW and TE. Therefore, we evaluated a ratio of the SI inside the stent to the image noise around the stent (IS/N) which was our original index. The IS/N was calculated using the subtracted images (obtained by subtracting an image series A from a different series B that was acquired using the same parameters) with the following equations:

(4) $$\text{IS}/\text{N}=\frac{1}{\sqrt{2}}\times \frac{{\text{SI}}_{\text{stent},\text{avg}}}{{\text{SD}}_{\text{subtra}}}$$

where

(5) $${\text{SI}}_{\text{stent},\text{avg}}=\frac{{\text{SI}}_{\text{stent},\text{A}}+{\text{SI}}_{\text{stent},\text{B}}}{2},$$

SI_stent, A_ and SI_stent, B_ indicate the SIs inside the stent adjacent to the coil (Fig. 2), measured from image series A and B, respectively. SD_subtra_ indicates the standard deviation obtained from a region of interest with 140 × 140 pixels that was centered on the subtracted transverse image. The IS/N was measured using ImageJ 1.48v (US National Institutes of Health, Bethesda, Md., USA).

These values (ALN, RIS_S_, RIS_C_, and IS/N) were measured for each of the consecutive seven slices that the coil was depicted on, and were measured for each value of the different imaging parameters. In other words, 14 values (seven slices for each of the image series A and B) of ALN, RIS_S_, and RIS_C_ each were obtained for each of the 26 parameters ([6 FAs + 7 BWs] × 2 scanners). On the other hand, seven IS/N values were obtained for each of the 26 parameters because a single IS/N value was measured by using a pair of image series A and B (subtraction images).

### Statistical analysis

One-way repeated measures analysis of variance and multiple comparisons (the Bonferroni method) were used to determine statistically significant differences (*P* < 0.05) in each of ALN, RIS_S_, RIS_C_, and IS/N between different FAs and BWs. The significant differences in each index between different values of FS (1.5T vs. 3.0T) were tested using the paired *t* test. These statistical analyses were conducted using the R version 3.2.1 (R Foundation for Statistical Computing, Vienna, Austria).

## Results

### Flip angle

**Fig 3.**
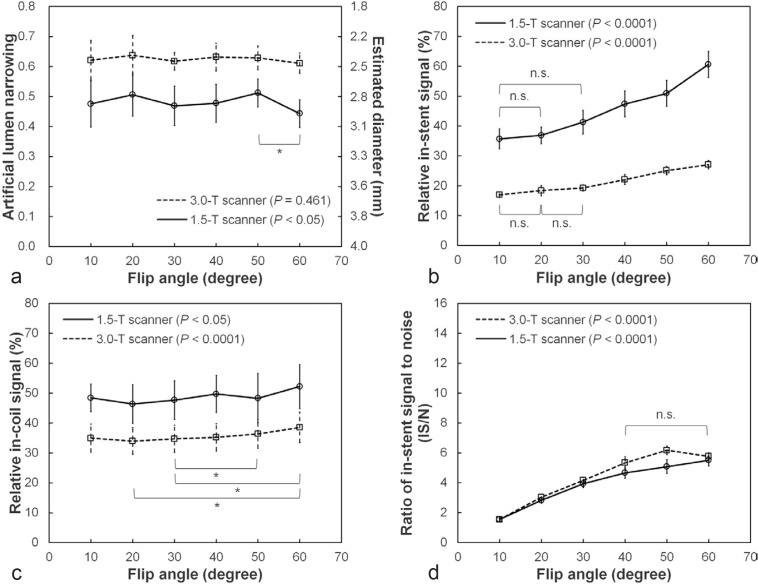
The effects of the flip angle on (**a**) the artificial lumen narrowing, (**b**) the relative in-stent signal, (**c**) the relative in-coil signal, and (**d**) the ratio of in-stent signal to noise (IS/N). The right y-axis in (**a**) represents the in-stent luminal diameter estimated from the A_stent_ in Eq. (1). The solid and dashed lines indicate the 1.5T and 3.0T MR scanners, respectively. Plotted values and error bars indicate the mean values and standard deviations, respectively, measured from 14 slices for (**a**–**c**) and 7 slices for (**d**). All the four indices showed significant variation (tested by the analysis of variance) with variation in flip angle, except for the ALN of the 3.0T scanner, and the *P* values are shown next to the figure legend (i.e., “1.5T scanner” or “3.0T scanner”). In (**b**) and (**d**), “n.s.” means “not significant” and the other pairs (no annotations) mean “significant differences” tested using multiple comparisons. In (**a**) and (**c**), the asterisks (*) mean significant differences and the other pairs (no annotations) mean “not significant”. Significant differences between the 1.5T and 3.0T scanners were observed for all four indices.

**Fig 4.**
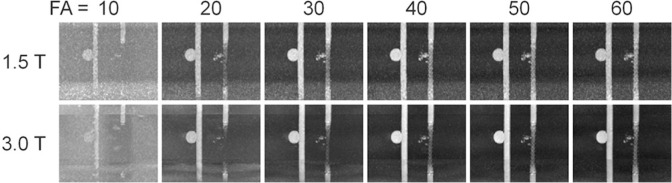
The maximum intensity projection images representing the effects of the flip angle (degree) and MF strength on visual image quality. The phantom with the stent and coil and the phantom without them are presented on the right and left, respectively, of each image.

The RIS_S_ increased significantly with an increase in FA (up to 60°) for both the 1.5T and 3.0T scanners (Fig. 3b), especially when the FA was higher than 30°. About 65% increase in RIS_S_ was observed at both values of FS when the FA was changed from 10° (RIS_S_ = 36% at 1.5T and 17% at 3.0T) to 60° (RIS_S_ = 61% at 1.5T and 27% at 3.0T), and the variation in RIS_S_ was maximum (the rate of variation of 19% [10 percentage points]) between 50° and 60° (RIS_S_ = 51% and 61%) at 1.5T. The variation in the RIS_S_ was visible on the MRA images (Fig. 4). The variations in ALN and RIS_C_ with varied FA did not show a particular trend (Fig. 3a and 3c). The IS/N increased with an increase in FA up to 50° and 60° for the 3.0T and 1.5T scanners, respectively (Fig. 3d). The maximum IS/N was about three times higher than the minimum for both scanners (max./min. = 5.5/1.6 at 1.5T and 6.2/1.6 at 3.0T).

### Bandwidth and echo time

**Fig 5.**
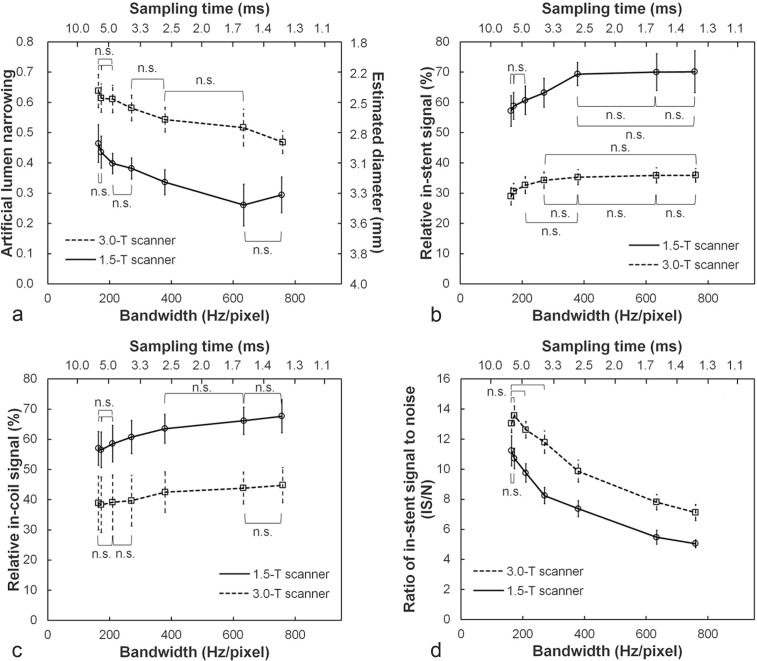
The effects of bandwidth and echo time on (**a**) artificial lumen narrowing, (**b**) relative in-stent signal, (**c**) relative in-coil signal, and (**d**) the ratio of in-stent signal to noise (IS/N). The right y-axis in (**a**) represents the in-stent luminal diameter estimated from the A_stent_ in Eq. 1. The top x-axes represent the sampling time calculated by an inverse of the bandwidth. The solid and dashed lines indicate the 1.5T and 3.0T MR scanners, respectively. Plotted values and error bars indicate the mean values and standard deviations, respectively, measured from 14 slices for (**a**–**c**) and 7 slices for (**d**). All four indices showed significant difference (tested by the analysis of variance, *P* < 0.0001 for all scanners and indices) between different bandwidths. “n.s.” means “not significant” and the other pairs (no annotations) mean “significant differences” tested using multiple comparisons. All four indices showed a significant difference between the 1.5T and 3.0T scanners.

**Fig 6.**
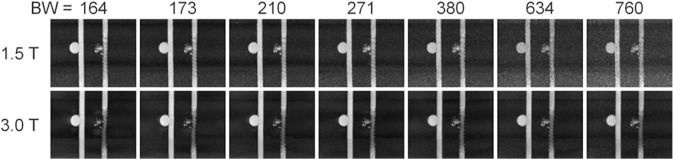
The maximum intensity projection images that represent the effects of bandwidth (Hz/pixel) and magnetic field strength on visual image quality. The phantom with the stent and coil and the phantom without them are presented on the right and left, respectively, of each image.

The ALN showed a lower value (a larger luminal area inside the stent) when a wider BW (shorter TE) was set (Fig. 5a), which was visible on the MRA images (Fig. 6). The difference in the estimated luminal diameter between the maximum and minimum ALNs was about 0.51 mm at both values of FS (max./min. = 3.35 mm/2.93 mm at 1.5T and 2.92 mm/2.41 mm at 3.0T). The ALN varied significantly with a variation in sampling time (an inverse of BW [Hz/pixel]) of at least 2 ms (*i.e.*, a variation of 2 data points in BW; Fig. 5 and Table 1). The RIS_S_ and RIS_C_ slightly increased with an increase in BW (Fig. 5b and 5c). However, sampling-time variations of 3 and 2 ms (variations of 3 and 2 data points) were required to obtain statistical differences in RIS_S_ and RIS_C_, respectively. The IS/N increased with a decrease in BW and the increase in TE (Fig. 5d). The maximum IS/N was about twice as high as the minimum (max./min. = 11.2/5.0 at 1.5T and 13.6/7.1 at 3.0T).

### Magnetic field strength

The 1.5T scanner indicated significantly better luminal visibility (29% lower ALN and two times higher RIS_S_ on average) and better delineation inside the coil (40% higher RIS_C_ on average) than the 3.0T scanner, regardless of the other parameters (Figs. 3a–c and 5a–c). Even the worst values (the lowest RIS_S_ and RIS_C_ and the highest ALN) of the 1.5T scanner were better than the best values (the highest RIS_S_ and RIS_C_ and the lowest ALN) of the 3.0T scanner (Figs. 3a–c and 5a–c). In other words, the differences in the ALN, RIS_S_, and RIS_C_ between the 1.5T and 3.0T scanners were larger than the variations due to the other parameters (FA, BW, and TE). On the other hand, the IS/N of the 1.5T scanner showed just a 17% lower value than that of the 3.0T scanner on average (Figs. 3d and 5d). The difference in the IS/N between the two scanners was smaller than the variations due to the other parameters.

## Discussion

The present study investigated *in vitro* the effects of imaging parameters on the image noise (IS/N in this study) and severity of artifacts on CE-MRA images of a cerebral aneurysm with a stent and coil. A few studies have investigated the relationship between imaging parameters and stent- or coil-induced artifacts.^5–7^ However, they did not take into account the image noise required for good image quality. Therefore, we believe that our results could help optimize imaging parameters in clinical practice.

An increase in FA led to significant increases in the RIS_S_ and IS/N. However, the ALN and RIS_C_ were not significantly affected by the FA (Fig. 3). Although an increase in FA can prolong the scan time, the prolongation in this study was found to be a maximum of 4.0 s only (Table 1). Therefore, a higher FA (up to 60°) could be optimal for acquiring CE-MRA images of stent-assisted coil with minimal loss of image quality and prolongation of scan time. A wider BW (shorter TE) improved the ALN, RIS_S_, and RIS_C_ (Fig. 5) and shortened the scan time (Table 1). However, the IS/N was worsened by a wide BW. In other words, the variation in BW necessitated a trade-off between IS/N and artifact severity. We cannot determine the best BW for CE-MRA of stent-assisted coil because the acceptable image quality would differ among medical facilities. Nevertheless, our quantitative assessment of the effect of BW on image quality could facilitate the determination of the optimal BW at every facility. The 1.5T MR scanner indicated better ALN, RIS_S_, and RIS_C_ than the 3.0T scanner, and FS had the maximum effect on artifact severity compared with the other parameters. In contrast, the 1.5T scanner indicated lower IS/N than the 3.0T scanner, although the effect of FS on IS/N was relatively smaller than that of the other parameters. Therefore, use of a 1.5T scanner could be more beneficial than that of a 3.0T scanner in CE-MRA for patients with a stent-assisted coil.

We showed that the IS/N increased with an increase in FA although the FA should have less effect on the image noise. This was because the SI was increased by the increased FA, while the image noise was not changed. The IS/N for the 3.0T scanner showed the highest value for an FA of 50°, whereas for the 1.5T scanner, the IS/N was maximum at FA = 60°. This may be because the Ernst angles inside the stent could be near 50° for the 3.0T scanner and over 60° for the 1.5T scanner.

As mentioned in the introduction, there are two kinds of artifacts induced by a stent and coil: the susceptibility artifact and the RF-shielding artifact.^7,8^ The former can be improved by using a short TE, wide BW, low FS,^9^ and the latter can be decreased by using high FA and low FS.^10,11^ The previous studies focused on either a stent or coil, but not both. On the other hand, we used both a stent and coil, and the present study clarified that the effect of imaging parameters on the artifacts severity was not different between stent-assisted coiling and either a stent and coil. The ALN was affected by the BW (Fig. 5a), but not by the FA (Fig. 3a). These results imply that the ALN could measure only the susceptibility artifact induced by a stent, and not the RF-shielding artifact.

There are two limitations to this study. First, the absence of flow may reduce the applicability of our results in clinical practice. The presence of flow may change the results of this study because the in-flow effect increases the SI while turbulent flow decreases it. Nevertheless, our results will be helpful in determining the parameters for CE-MRA because our *in vitro* study clarifies the effect of the devices on image quality. Second, only one type of stent and coil were used in this study. Different types of devices could have different effects on image quality.

## Conclusion

A high FA and low field strength (1.5T MR scanner rather than 3.0T scanner) should be used for improved artifact severity and image noise in contrast-enhanced magnetic resonance angiography for a cerebral aneurysm treated using stent-assisted coiling. A wide BW (short TE) could decrease artifact severity at the expense of the image noise.

## Figures and Tables

**Table 1.** Imaging parameters used in this study

Series #	FS (Tesla)	FA (degree)	BW (Hz/pixel)	TS (ms)	TE* (ms)	TR** (ms)	Scan duration (s)
1	1.5	**10**	634	1.58	6.60	9.24	36.6
2	1.5	**20**	634	1.58	6.60	9.24	36.6
3	1.5	**30**	634	1.58	6.60	9.30	36.8
4	1.5	**40**	634	1.58	6.60	9.46	37.5
5	1.5	**50**	634	1.58	6.60	9.62	38.1
6	1.5	**60**	634	1.58	6.60	9.79	38.8
7	3.0	**10**	634	1.58	6.60	8.92	35.3
8	3.0	**20**	634	1.58	6.60	8.98	35.6
9	3.0	**30**	634	1.58	6.60	9.22	36.5
10	3.0	**40**	634	1.58	6.60	9.46	37.5
11	3.0	**50**	634	1.58	6.60	9.70	38.4
12	3.0	**60**	634	1.58	6.60	9.94	39.3
13	1.5	60	**164**	**6.10**	6.53	11.97	47.4
14	1.5	60	**173**	**5.78**	6.35	11.64	46.1
15	1.5	60	**210**	**4.76**	5.68	10.45	41.4
16	1.5	60	**271**	**3.69**	4.99	9.23	36.6
17	1.5	60	**380**	**2.63**	4.32	8.04	31.8
18	1.5	60	**634**	**1.58**	3.69	6.87	27.2
19	1.5	60	**760**	**1.32**	3.67	6.74	26.7
20	3.0	60	**164**	**6.10**	6.45	12.12	48.0
21	3.0	60	**173**	**5.78**	6.28	11.79	46.7
22	3.0	60	**210**	**4.76**	5.63	10.63	42.1
23	3.0	60	**271**	**3.69**	4.97	9.43	37.3
24	3.0	60	**380**	**2.63**	4.34	8.83	35.0
25	3.0	60	**634**	**1.58**	3.82	8.83	35.0
26	3.0	60	**760**	**1.32**	3.76	8.83	35.0

BW, bandwidth; FA, flip angle; FS, magnetic field strength; TE, echo time; TR, repetition time, TS, sampling time. The **bold figures** represent the different values of the parameters that were varied in the experiment.

* TE automatically changed depending on BW because TE was set at “Shortest” for series #13–26.

** TR automatically changed depending on BW and FA because TR was set at “Shortest” for all series.
